# Subcellular and *in-vivo* Nano-Endoscopy

**DOI:** 10.1038/srep34400

**Published:** 2016-10-03

**Authors:** Surya Venkatasekhar Cheemalapati, John Winskas, Hao Wang, Karthik Konnaiyan, Arseny Zhdanov, Alison Roth, Swamy Rakesh Adapa, Andrew Deonarine, Mark Noble, Tuhin Das, Robert Gatenby, Sandy D. Westerheide, Rays H. Y. Jiang, Anna Pyayt

**Affiliations:** 1 IBIS Lab, Department of Chemical and Biomedical Engineering, University of South Florida, Tampa, FL 33647, USA; 2 Department of Global Health, College of Public Health, University of South Florida, Tampa, FL 33647, USA; 3 The Department of Cell Biology, Microbiology and Molecular Biology, College of Arts and Sciences, University of South Florida, Tampa, FL 33620, USA; 4 Departments of Radiology and Integrated Mathematical Oncology, Moffitt Cancer Center, Tampa FL 33612, USA

## Abstract

Analysis of individual cells at the subcellular level is important for understanding diseases and accelerating drug discovery. Nanoscale endoscopes allow minimally invasive probing of individual cell interiors. Several such instruments have been presented previously, but they are either too complex to fabricate or require sophisticated external detectors because of low signal collection efficiency. Here we present a nanoendoscope that can locally excite fluorescence in labelled cell organelles and collect the emitted signal for spectral analysis. Finite Difference Time Domain (FDTD) simulations have shown that with an optimized nanoendoscope taper profile, the light emission and collection was localized within ~100 nm. This allows signal detection to be used for nano-photonic sensing of the proximity of fluorophores. Upon insertion into the individual organelles of living cells, the nanoendoscope was fabricated and resultant fluorescent signals collected. This included the signal collection from the nucleus of Acridine orange labelled human fibroblast cells, the nucleus of Hoechst stained live liver cells and the mitochondria of MitoTracker Red labelled MDA-MB-231 cells. The endoscope was also inserted into a live organism, the yellow fluorescent protein producing nematode Caenorhabditis elegans, and a fluorescent signal was collected. To our knowledge this is the first demonstration of *in vivo*, local fluorescence signal collection on the sub-organelle level.

Nano-scale instruments that allow sensing at the cellular and subcellular level^1^,^2^,^3^ are crucial for gaining a fundamental understanding of the molecular dynamics inside the single cell. It is likely this information will help elucidate the pathophysiology of many diseases such as cancer and Alzheimer’s^4^,^5^,^6^,^7^ and facilitate the design of new therapies^1^,^8^,^9^. Various single cell devices have been reported including carbon nanotubes on Atomic-force microscope (AFM) tips^10^,^11^; glass pipettes^5^,^12^; capillaries^13^; boron nitride nanotubes affixed onto tungsten wires^14^; sharpened macroscopic needles^15^; Ag nanowires on electrochemically etched Tungsten tip^16^; Focused ion beam (FIB)-milled AFM tips^17^; optical fibre-nanowire-based endoscopes^18^,^19^; optical fibre-photonic crystal based endoscope^20^; as well as, nanofibres nanofibres^6^,^9^,^21^,^22^,^23^,^24^,^25^,^26^,^27^,^28^. These devices can perform the intracellular sensing of enzymes^6^,^21^, pH^22^, calcium ions^9^,^23^, carcinogens^26^, biomarkers^24^,^25^,^27^, and quantum dots^18^ using fluorescence. Intracellular proteins can be investigated using photoluminescence^20^ or localized surface plasmon resonance^28^. *In-situ* electrochemical measurements^12^,^14^ or Surface-enhanced Raman spectroscopy (SERS) measurements for sensing biomolecules in single cell organelle^12^,^13^,^29^ can also be conducted. Apart from sensing, these devices may also be used to perform the payload delivery of quantum dots^10^,^15^,^18^, DNA^17^, and other materials^12^. Widespread application of these devices has been limited due to fabrication issues and their need for manual assembly^18^,^20^, and they usually require sophisticated external detectors for sensing^30^.

This method creates the nanoscale-endoscope that is easily fabricated in large quantities, is minimally invasive, allows for single cell manipulation, and has the capability to simultaneously transmit and receive optical signals from intracellular organelle without the use of external detectors. This technology permits the efficient and cost effective *in vitro* and *in vivo*, single cell organelle analysis. The experiment involved FDTD simulations of light emerging from a tapered nanoendoscope tip and simulations of the back coupling of light into the nanoendoscope taper. Multiple nanoendoscopes were fabricated and characterized using scanning electron microscopy. The Experimental validation of forward and back coupling of light into the endoscope was obtained by collecting optical signals from fluorescently labelled intracellular organelle of fibroblast, liver cell line and MDA-MB-231cancer cells. These experiments demonstrated that fluorescent light can be coupled back through an endoscope and the localized spectra from the cell interior can be acquired. This advancement allowed for the intracellular fluorescent spectrum to be collected *in-vivo* from live Caenorhabditis elegans worms.

## Results and Discussion

**Figure 1:**
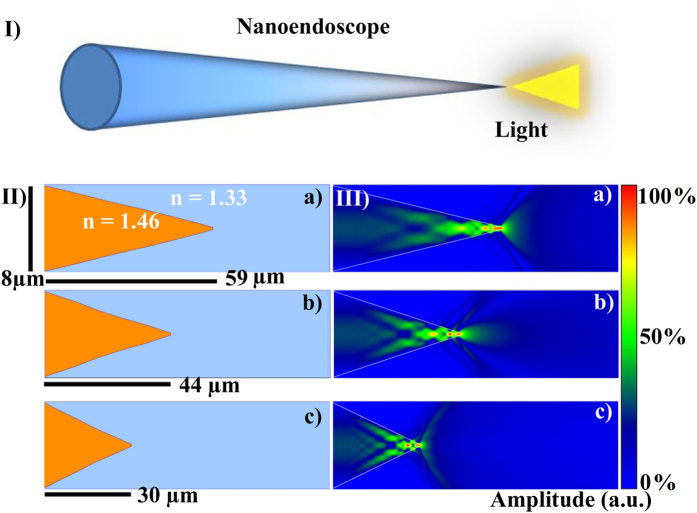
Light coupling into nanoscale endoscope. (I) Schematic of a nanoendoscope showing light shining out of the tip. (II,III) FDTD simulations of light coupling into nanoendoscopes tapered from 8 μm to 200 nm over different lengths (a) 59 μm (b) 44 μm and (c) 30 μm.

Figure 1(I) shows the schematic of the nanoendoscope that can guide light and can be inserted safely into single cells for detailed spectrum analysis, both *in-vitro* and *in-vivo*. Figure 1(II,III) shows FDTD simulations of light transmission profiles, with tapers consisting of an 8 μm base narrowing to a 200 nm tip over taper lengths ranging from 30 μm to 59 μm. It is observed that the shape of the output beam is a function of the taper length. At a taper length of 30 μm, the transmission of light was very local and close to the apex. For the taper lengths of 44 μm and 59 μm, the mode propagating in the optical fibre was shown to be continuously squeezed along the taper of the nanoendoscope, before fanning out at the apex. For the 44 μm taper length, the transmission is focused over a distance of several micrometers from the apex; comparatively, the 59 μm taper length emits an unfocused beam that spreads widely at a very short distance from the apex. The range of light transmission geometries allows for different tapered profile tips to be used for different application. For example, a taper length of 30 μm can be utilized for an experiment that requires near field excitation, while taper lengths of 44 μm or 59 μm can be used for local fluorescent spectra collection inside single cells.

**Figure 2:**
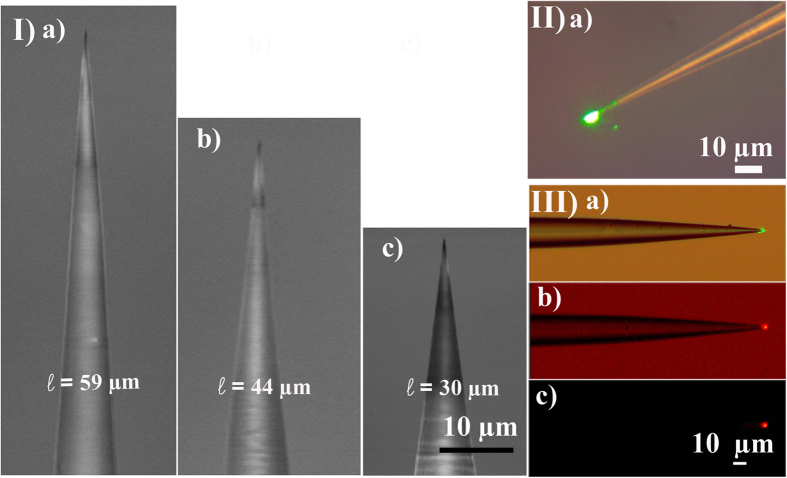
Nano endoscope characterization. Part I shows scanning electron microscope images of the single cell endoscope tips fabricated with different tapered lengths (a) 59 μm (b) 44 μm and (c) 30 μm. Part II) (a) experimental demonstration of 532 nm light coupling into the endoscope under microscope for 44 μm tapered length fibre. Part III is excitation of fluorescent dye on the endoscope tip using 532 nm light, (a) is green light coupling to the endoscope, (b) is excitation of dye observed using red filter with ambient light turned on and (c) is excitation of dye observed using red filter with ambient light turned off.

Using conventional (HF) wet etching technique^31^,^32^, nanoendoscope tips of the three lengths corresponding to the simulations, 30 μm, 44 μm and 59 μm were fabricated. This process was shown to be reproducible by using scanning electron microscopy to characterize the results of the wet etch processing. Figure 2I shows nanoendoscope tips of different taper lengths, ranging from 30 μm to 59 μm with a nanoscale apex diameter close to 200 nm. The fabricated nanoendoscopes were fusion spliced to the cleaved end of an optical fibre and inserted to an Eppendorf micro manipulator for nm resolution control of nanoendoscope movement during experiments. Figure 2II shows optical microscopy images of 532 nm light shining out of the nanoendoscope tip. The shape of the beam was very similar to the simulated shown in Fig. 1III(b) and the light propagates over a distance of several micrometers in agreement with the simulations. Figure 2III shows the excitation of a fluorescent dye when the nanoendoscope forward coupled with 532 nm light was dipped into the dye solution. It can be observed that the nanoendoscope is able to locally excite fluorescence in the solution.

**Figure 3:**
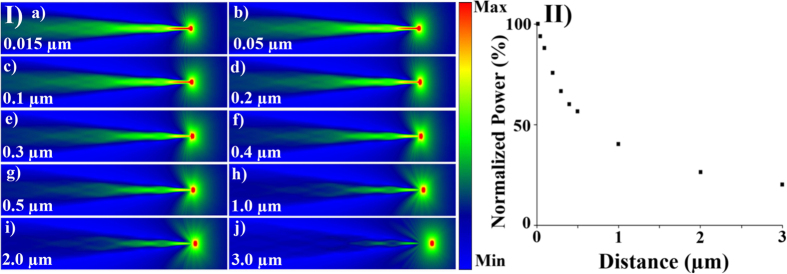
Nanoendoscope tip light collection characterization. Part I (a) shows FDTD 2D single cell endoscope tip tapering from an 8 μm diameter base to 200 nanometers at the apex of the tip over a distance of 44 μm with a point light source places at different distances from the tip from 0.015 μm to 3.0 μm. (II) Is the plot of normalized power (%) with respect to 0.015 μm distance from the point source to the apex of optical fibre.

Figure 3I(a–j) shows the back coupling of light into a nanoendoscope with the taper length 44 μm from a point source located at different distances from the apex of the tip. It is observed that the coupling efficiency quickly decreases when the point source is moved from 0.015 μm to 3.0 μm away from the apex of the nanoendoscope tip. This information can be applied to a case when an external light source is used for the excitation of a fluorescent sample and a nanoendoscope is used to collect the intensity of the fluorescent signal. The relative intensity decreases from 100% at distance 0.015 μm from the apex to 40% over a distance of 1 μm, Fig. 3II. In order to simulate the simultaneous forward and back coupling of light and to optimize the geometric position of the fluorescent source with respect to the endoscope tip for effective back coupling, further analysis was conducted.

**Figure 4:**
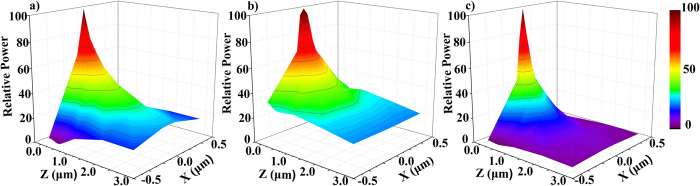
Theoretical optimization of light delivery and collection by the nanoscale endoscope tip. (**a**) Forward coupling of light (**b**) Back coupling of light (**c**) is a product of forward and back coupling intensities.

Figure 4 shows 3D plots of the relative power intensities for (a) forward (b) back and (c) the cross product of the forward and backward coupling. The Z axis is parallel to the endoscope. The X axis is perpendicular to the direction of light propagation. Intensity of light transmitted in the endoscope is shown in Fig. 4(a). Efficiency of coupling from different points (X, Z) into the endoscope tip is shown in Fig. 4(b). The third coordinate in the images represents light intensity. It can be observed that light coupled into endoscope illuminates microscale area. Light intensity drops to 30% 2 μm away from the tip. In Fig. 4(b) back-coupling efficiency is also local, and only 20% of the signal can be detected 3 μm away from the tip. In order to simulate simultaneous forward and back coupling of light, the product of forward and backward intensities is calculated and shown in Fig. 4(c). The total collection efficiency drops to 45% after moving by just 300 nm away from the tip. This demonstrates that when nanoendoscope is used for light delivery, the collection by the same nanoendoscope tip is very local and works efficiently only within 100’s of nanometers. Thus detection of fluorescent signal combined with simultaneous delivery can be used for nanophotonic sensing of XYZ position of the fluorophore. The local signal detection in proximity to fluorescent dye was demonstrated using simultaneous light delivery and collection in Supplementary Information. Next *in vitro* and *in vivo* light collection experiments were conducted.

First, three types of cell viability experiments were conducted and reported in the Supplementary Information demonstrating that the device can safely penetrate single cells without short term or long term damage. Following this, spectrum collection from a number of commercially available florescent dyes using external excitation and simultaneous excitation and emission were conducted to demonstrate broad spectral range that can be used.

**Figure 5:**
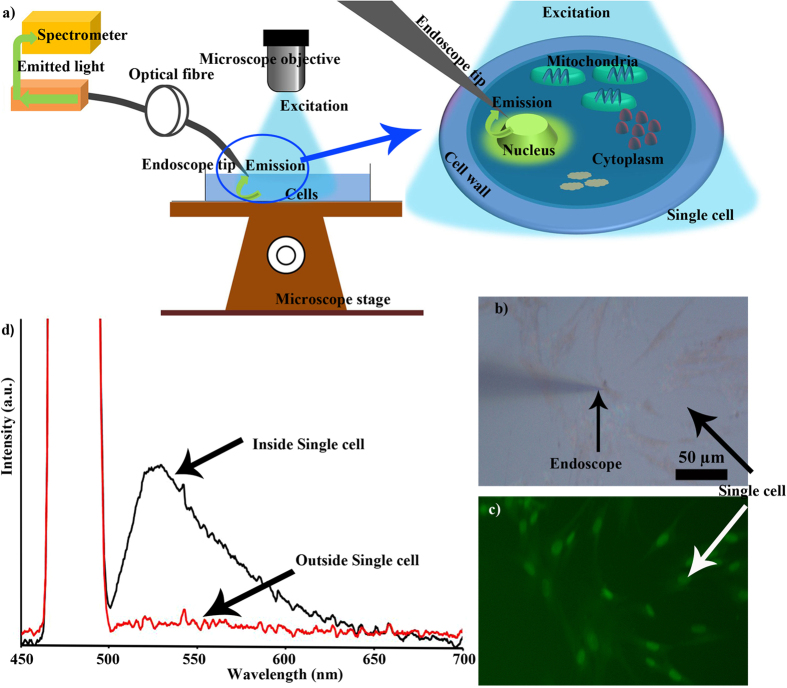
*In vitro* spectrum collection using nanoendoscope. (**a**) Schematics of the experiment where internal microscope light source was used for fluorescence excitation. (**b**) A bright field image of the nanoendoscope inserted into a fibroblast single cell and (**c**) the corresponding fluorescence image. (**d**) The black curve is the spectrum (peak ~525 nm) from a single cell collected using nanoendoscope, while red curve is the spectrum taken outside the cell. The Y axis shows intensity (linear scale).

Figure 5 summarizes the fluorescence spectra obtained from the excitation of a single fibroblast human lung cell stained with Acridine Orange. Figure 5(a) shows the schematics of the experiment. The nanoendoscope was inserted into the cell using a high precision 40 nm step resolution micromanipulator that was controlled using custom made software. The cell nuclei were labelled with Acridine Orange which has an absorption peak at 490 nm and a fluorescence emission peak at around 525 nm. The cells were excited with blue light generated by passing the microscope broadband illumination light via fluorescent filter. As the cell emitted the fluorescence signal from its interior, the endoscope at a close proximity to the organelle (Fig. 5(b,c)) collected the spectrum. Figure 5(d) shows the emission peak (black) at 525 nm for Acridine orange in a single cell. While outside of the cell (red curve), no signal at 525 nm was detected. Here we have demonstrated the nanoendoscopes ability to collect the spectrum from sub-cellular organelle.

**Figure 6:**
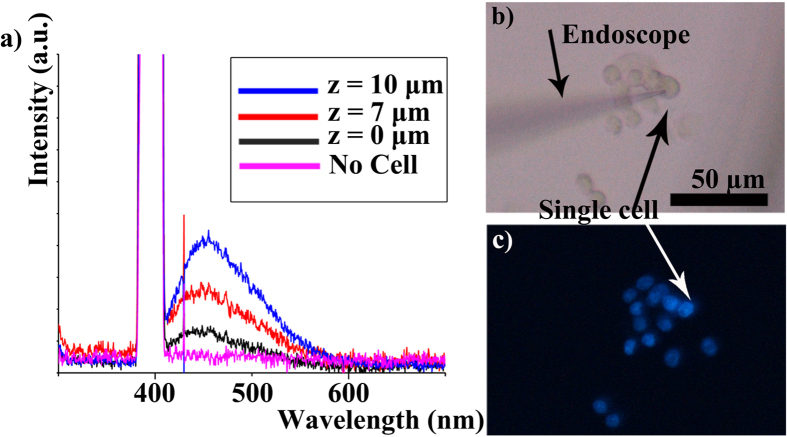
*In vitro* spectrum collection using nanoendoscope. (**a**) Spectrum collected from Hoechst stained cells at different heights in the cell. (**b**) A bright field image of the nanoendoscope inserted into a Live liver cell (**c**) the corresponding fluorescent image.

Next, live liver cells were stained with Hoechst (see details in Supplementary Information) and probed with a nanoendoscope (Fig. 6). Spectra were taken as the nanoendoscope was inserted into the nucleus of the cells (Fig. 6(a,b)). It can be observed in Fig. 6(a) that the fluorescence intensity increased as nanoenscope goes deeper into the interiors of the cell. “No cell” indicates background signal, z = 0 μm is when endoscope initially penetrates the cell surface, and z = 7 and 10 μm are signals obtained when nanoendoscope goes deeper into the cell by these distances. This difference cannot be perceived using conventional optical microscopy as seen in Fig. 6(c). The change in spectra closely resembles that observed in FDTD simulations. Spectral information with respect to the location of the nanoendoscope can be used for high resolution imaging or to locate intracellular organelle.

**Figure 7:**
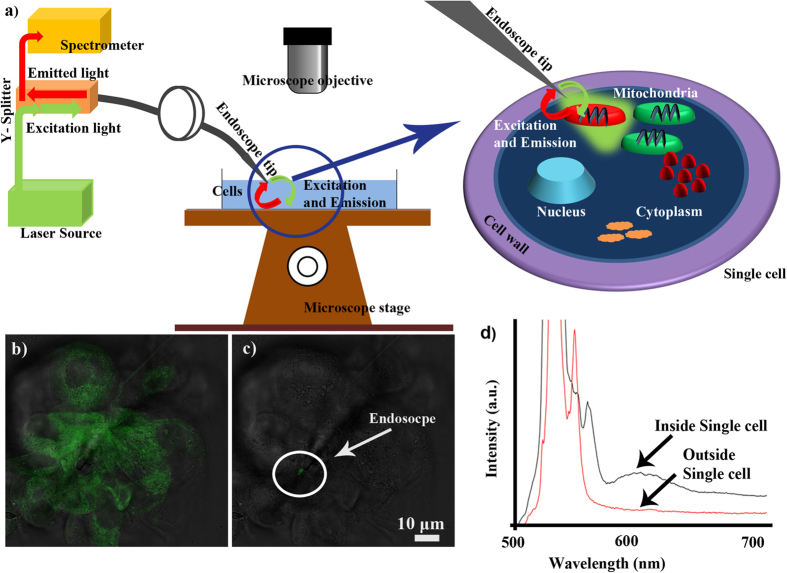
Fluorescent spectrum collected using configuration shown in (**a**) from a Mito tracker stained MDA-MB-231 cell. (**b**) Fluorescent image observed under microscope when entire field of view was illuminated by excitation light, and (**c**) fluorescent image when local fluorescence was excited with the nanoendoscope. (**d**) Spectra from inside a Mito-tracker-labeled cell (peak ~600 nm) and outside the cell.

Next, an experiment for the simultaneous delivery and collection of optical signals via the single cell nanoendoscope was conducted on live MDA-MB-231 cells that were stained with mito tracker (Fig. 7(a)). The staining procedure is provided in the Supplementary Information. The expected value for excitation and emission were approximately 525 nm and 600 nm respectively. The excitation source, a 532 nm green laser, was coupled into the endoscope via a Y splitter. Figure 7(b) shows fluorescent image of the whole cell exposed to wide field excitation. Figure 7(c) demonstrates local excitation of fluorescence using nanoscale endoscope delivering light into the cell. The fluorescence emitted near the nanoendoscope tip was coupled back and analyzed using spectrometer (Fig. 7(d), black curve).

**Figure 8:**
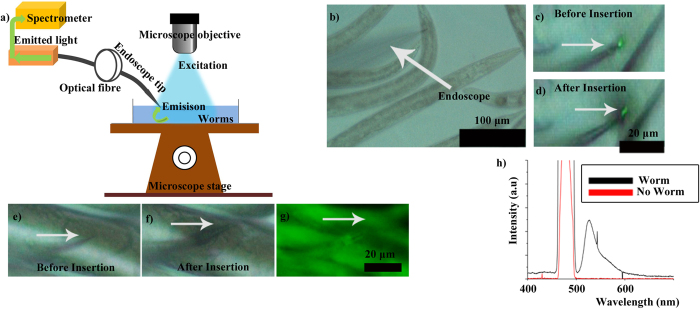
*In-vivo* fluorescent signal collection from a live *C. elegans* nematodes (AM140). (**a**) The schematics of the experiment. (**c**–**f**) Optical microscopy images showing nanoendoscope during insertion into the worm body (**c,d**) and an egg (**e,f**). (**g**) Fluorescence of the worm observed under a microscope with corresponding spectrum (black curve) collected via nanoendoscope (**h**), where “no worm” (red) indicates the background signal without worm.

Next, *in vivo* experiments were conducted using *C. elegans* strain AM140, which harbors an integrated multi-copy neurodegeneration model transgene integrated into the genome^33^. The sample preparation is provided in the Supplementary Information. The worms fluoresce upon excitation with blue light and emit green signal at ~525 nm. The nanoendoscope was inserted into the worms as shown in Fig. 8(a–f). Yellow fluorescent protein (YFP) in the worms was excited using an external microscope light (Fig. 8(g)) and a spectrum was collected using the nanoendoscope. The spectrum from the *in vivo* experiment shows a peak (black curve) at 525 nm (Fig. 8(h)).

## Conclusion

A single cell endoscope that can simultaneously deliver and collect light on the sub-cellular level was fabricated. FDTD simulations of simultaneous light delivery and collection showed that the performance of endoscope depended on the location of the tip with respect to the fluorescent sample. Following the simulations, the endoscope was shown experimentally to collect the spectrum from intracellular organelle in two configurations, using an external light source and light source propagating from the endoscope itself. We were also able to insert nanoendoscope and measure fluorescence in a live multicellular organism. To our knowledge, this is the first time a nanoendoscope has been used to collect *in-vivo* fluorescence spectrum. This technology is an important step towards the realization of *in vivo* analysis that eliminates the requirement of a microscope objective for signal collection.

## Methods

The nanoendoscope taper profile optimization was conducted using FDTD simulations in commercially available software OPTI-FDTD. A silicon dioxide fibre with a refractive index of 1.46 was modeled to have taper with a diameter ranging from 8 μm base to 200 nm tip over a taper length of 30 μm, 44 μm and 59 μm (Fig. 1). Green light, with a wavelength of 532 nm, was coupled into each respective structure and subsequently the shape of the outcome beam was analyzed.

Next, back coupling simulations were conducted by placing a point source of light near the tip of the nanoendoscope and relocating the source in a 2D array of points to investigate efficiency of coupling at the different geometric locations. This was used to approximate the mechanisms and properties used when collecting fluorescence from a sample excited by an external light source. Further analysis was then conducted to investigate the efficiency of the back coupling of light if measured while forward coupled light was simultaneously propagating through the endoscope.

Following this, single cell nanoendoscopes of three different taper lengths 30 μm, 44 μm and 59 μm were fabricated from cleaved single mode silicon dioxide 125/8 μm fibres using HF wet etching^31^,^32^. The resultant tapered fibre tips were characterized using a HITACHI SU-70 scanning electron microscope (SEM) and then spliced to a regular single mode fibres using an IFS 10 Arc fusion splicer to provide an optical connection with minimal loss. Several nanoendoscope viability tests have been conducted on single cells and reported in Supplementary Information.

After these simulations, experiments that measured the light collected from sub cellular organelle via the single cell nanoendoscope were conducted using two experimental setups. In the first setup, a human lung fibroblast cell was stained with Acridine orange and excited using an external light source generated by a built-in Nikon, blue excitation, fluorescent filter. The fluorescent emission signal was then collected using the nanoendoscope which had been inserted into nucleus of the cell. Similarly, signal was collected from Hoechst stained Liver cells. In the second setup, the nanoendoscope was inserted into an MDA-MB-231 cell that was stained with MitoTracker Red. The fluorescence in the cell was excited using green light (532 nm) forward coupled into the nanoendoscope. Simultaneously, the back coupled fluorescent emission signal was collected and measured using the same nanoendoscope. Following this, *in-vivo* experiments were conducted using *C. elegans* worms. Neurodegeneration model animals that stably express polyglutamine residues fused to Yellow Fluorescent Protein (YFP) in the body wall muscle (strain AM140, genotype rmIs132 unc-54p::Q35::YFP)^33^, were anesthetized with 10 mM Levamisole, mounted on 2% agarose pads, and then probed with the nanoendoscope. The emitted spectrum was then collected by the nanoendoscope.

## Additional Information

**How to cite this article**: Cheemalapati, S. V. *et al*. Subcellular and *in-vivo* Nano-Endoscopy. *Sci. Rep.* **6**, 34400; doi: 10.1038/srep34400 (2016).

## Supplementary Material

Supplementary Information
