# Supplementary material for: Subcellular and in-vivo Nano-Endoscopy
Source: Sci Rep. 2016 Oct 3;6:34400. doi: 10.1038/srep34400 (PMC5046067; doi:10.1038/srep34400)
Supplement: Supplementary Information [file srep34400-s1.pdf]

Subcellular and *in* -vivo Nano-Endoscopy

(Supplementary Information)

Surya Venkatasekhar Cheemalapati[1], John Winskas[1], Hao Wang[1], Karthik Konnaiyan[1], Arseny Zhdanov[1], Alison Roth[2], Swamy Rakesh Adapa[2], Andrew Deonarine[3], Mark Noble[3], Tuhin Das[4], Robert Gatenby[4], Sandy D. Westerheide[3] , Rays H.Y[2]. Jiang and Anna Pyayt[1].

[1]*IBIS Lab, Department of Chemical and Biomedical Engineering, University of South Florida, Tampa, FL 33647, USA*

[2] *The Department of Cell Biology, Microbiology and Molecular Biology, College of Arts and Sciences, University of South Florida, Tampa, FL 33620*

[3]*Department of Global Health, College of Public Health, University of South Florid, Tampa, FL 33647, USA*

[4]*Departments of Radiology and Integrated Mathematical Oncology, Moffitt Cancer Center, Tampa FL 33612, USA*

Correspondence and requests for materials should be addressed to AP (email: pyayt@usf.edu)

KEYWORDS Single cell analysis, sub-cellular spectroscopy, nanoscale, subcellular organelles, MDA-MB-231 cells, *Caenorhabditis elegans*

Cell viability Experiments

Cell viability is an important quality for the single cell analysis device [1]. The device can be called minimally invasive if a cell is able to survive the probing of nanoendoscope with a minimal damage. This means the device must be biocompatible and small. The cell viability can be tested using different methods. In [2] the cell was probed with the nanoendoscope and was continuously monitored using the optical microscope for its behavior. It was shown that the cell

had undergone mitosis after probing with the nanoendoscope thereby providing evidence of cell viability. In another study using photonic crystals [3], the nanobeam was probed in a population of cells using live-dead cell kit. Long term effects of the nanobeam was studied after it was cleaved inside the cell and was monitored the cell for several days. In another study [4], the calcium wave signalling due to mechanical disturbance caused by probing the cell with carbon nanotube endoscope was monitored. The signal was compared with those generated during probing with a glass pipette. It was shown that the carbon nanotube based nanoendoscope due to its small diameter induced less stress than the glass pipette.

In this study, three different types of viability experiments were conducted. In the first type, a nanoendoscope was placed inside the cell membrane and was kept in constant position while the cell was continuously monitored. In the second experiment, the nanoendoscope was placed inside the cell membrane similar to the previous experiment. Here, instead of leaving the nanoendoscope in a constant position, the probe was moved deep into the cell in precise steps at regular time intervals. In the third experiment, multiple cells were probed briefly and were monitored after 24 hour time interval to observe the long term effects.

Experiment 1

Figure S1 shows the bright field (a) and fluorescent microscopy images (b) and (c) of fibroblast cells that were probed with the nano endoscope. The cells were stained with live-dead cell kit. Live cells emit green light. The nanoenscope was inserted into the cells using micromanipulator and was kept in constant position. Fluorescent images were taken at 5 minutes intervals. Figure S1 (b) is the image taken at the beginning of the experiment (t=0 min). Figure S1 (c) is the image taken at 55 minutes from the start of the experiment (t=55 min). In the next cell viability study, the cell was constantly probed with the nano endoscope and monitored under optical microscope.

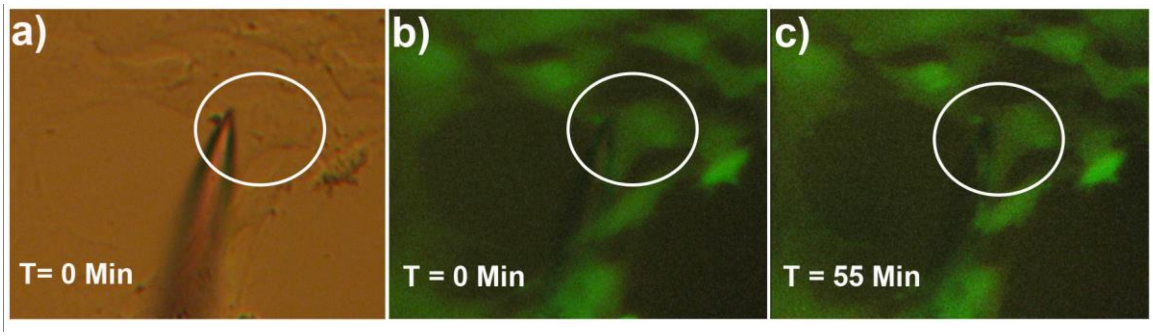

Figure S1 Investigation of cell viability with endoscope. a) Bright field image of endoscope inserted into single cell t = 0 b) corresponding fluorescent image at t = 0 c) fluorescent image of the cell at t = 55 minutes showing the cell is alive. The white circles indicate the cell inserted with the nanoendoscope.

Experiment 2

Figure S2 shows the bright field (a) and florescent images (b) of nano endoscope inserted into a liver cell. This kind of cell is on average 20 micrometer thick, as was shown using confocal microscopy on a similar population of cells. In Figure S2 I (a) and (b) live cells can be observed before the nanoendoscope was inserted. The labels (z) on the images indicate the current position of the nanoendoscope with respect to the final position. The cell was probed in Figure S2 II (a) and (b) at z = 10 μm, close to the cell surface. Next, in two successive steps, approximately 6 minute apart, the cell was penetrated deeper. First, the endoscope was inserted by 7 μm, Figure S2 III (a) and (b), and then, 3 μm deeper, Figure S2 IV (a) and (b), z = 0 μm. During the entire experiment, video was continuously recorded and filters were alternated between bright field and fluorescent field. All the images were extracted from the video. In the final position, Figure S2 IV (a) and (b) (z = 0 μm), the nanoendoscope was placed for next 30 minutes. Then the endoscope was removed, Figure S2 V (a) and (b), and cells were observed using fluorescent filter.

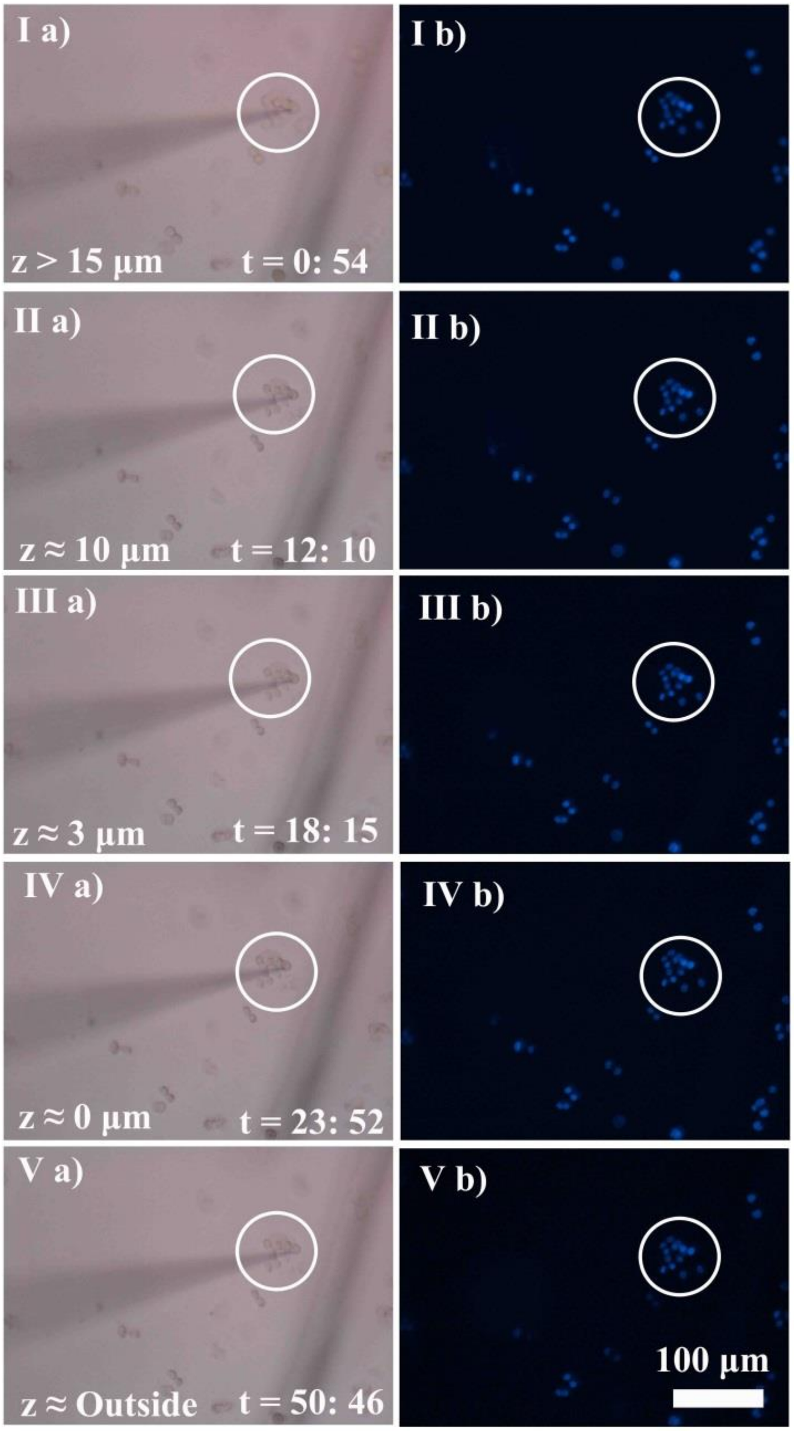

Figure S2 Continuous perturbation of liver cells with nano endoscope to demonstrate their long term viability.

Figure S3 shows fluorescent images taken before and after the experiment. The slight change in brightness of fluorescence is artifact related to automatic adjustment of the camera setting. The images look identical indicating minimal damage to the cells after continuous perturbation for nearly 50 minutes. It can be observed that the cells remained viable after the experiment. This demonstrates that sensing inside thick cells, where deep penetration is required, can be achieved.

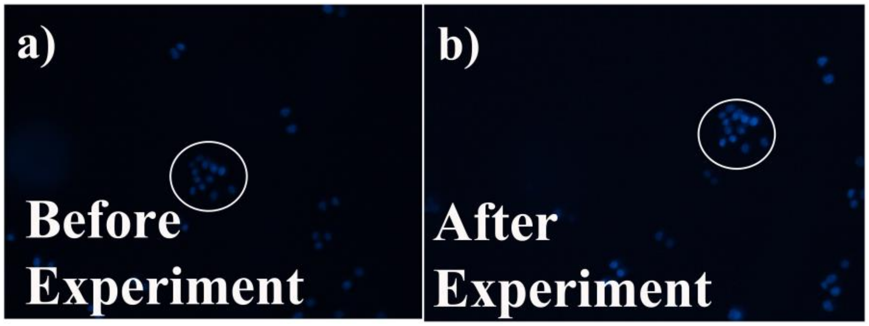

Figure S3 Hoechst stained liver cells observed before and after the perturbation experiment.

Experiment 3

In the next experiment multiple liver cells were probed once as shown in Figure S4 and analyzed after 24 hours to monitor any long term effects on these cells as shown in Figure S5. The cells were stained with Hoechst and cell tracker. Figure S5 shows a representative image of a cell (out of multiple cells) that was probed with the nanoendoscope. It can be observed that the cells were alive and healthy after 24 hours, their morphology looks good and they are fluorescent. This provides evidence that probing them once for a short time does not cause long term consequences. This also indicates that the same cells potentially can be probed multiple times. These experiments provide evidence that the nano endoscope is minimally invasive providing a platform for continuous monitoring or multiple analyses on single cells

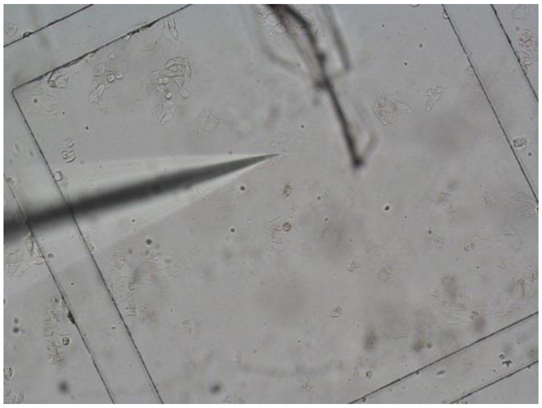

Figure S4 Probing cells with nano endoscope. Multiple cells were probed and observed for 24 hours.

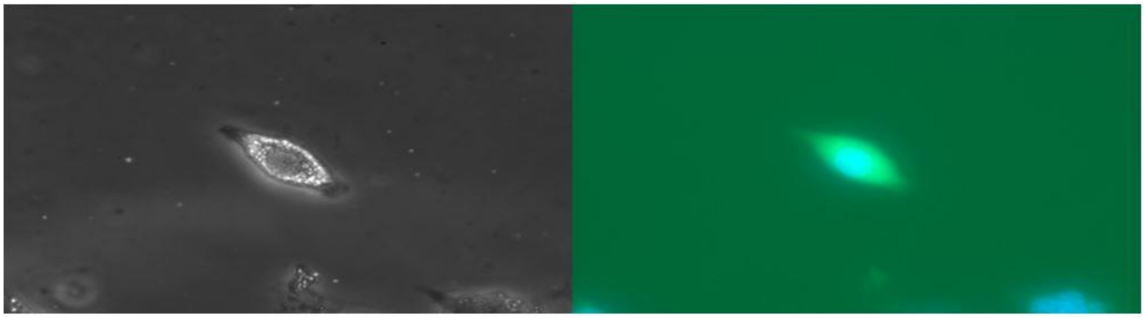

Figure S5 Representative picture of several cells observed for viability. (a) Bright field and (b) Fluorescent imaging of cells probed with the nanoendoscope as observed 24 hours after the probing.

Fluorescent Dye Experiments: External Excitation and Collection:

Spectrum collection using external excitation was conducted first on a group of commercially available florescent dyes. Five fluorescent dyes—Fluorescein, Green Fluorescent Protein, Bromfluorescein, Rhodamine 6G, and Rhodamine B, were used in the experiments. Small portable UV-emitting light source with a range of UV wavelengths was used for the excitation.

The endoscope was dipped into the solutions, and spectra were collected and analyzed using a spectrum analyzer.

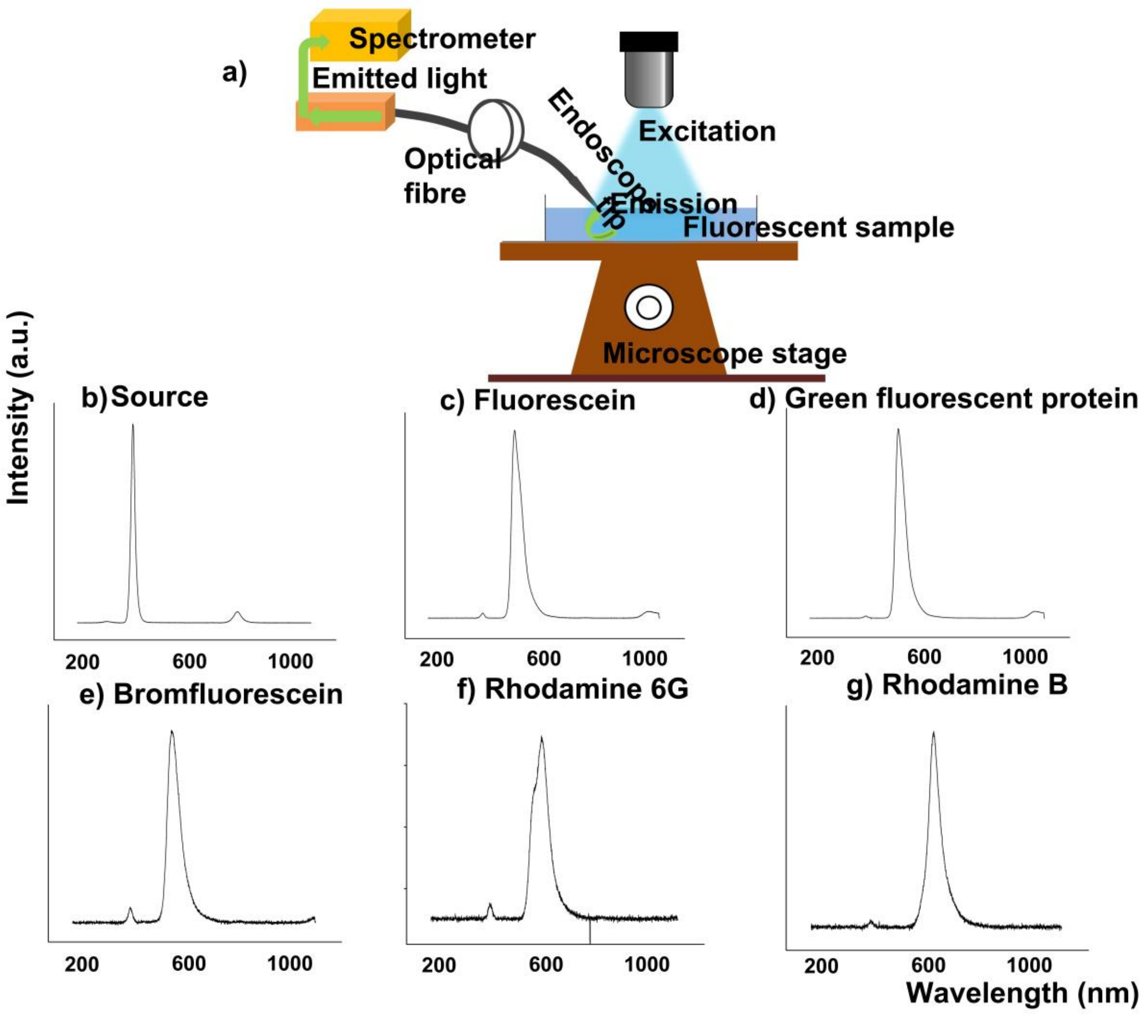

Figure S6 Light collection from fluorescent dyes. a) Experimental schematic of light collection experiments from multiple fluorescent dyes c-g. b) is the spectra of the external source.

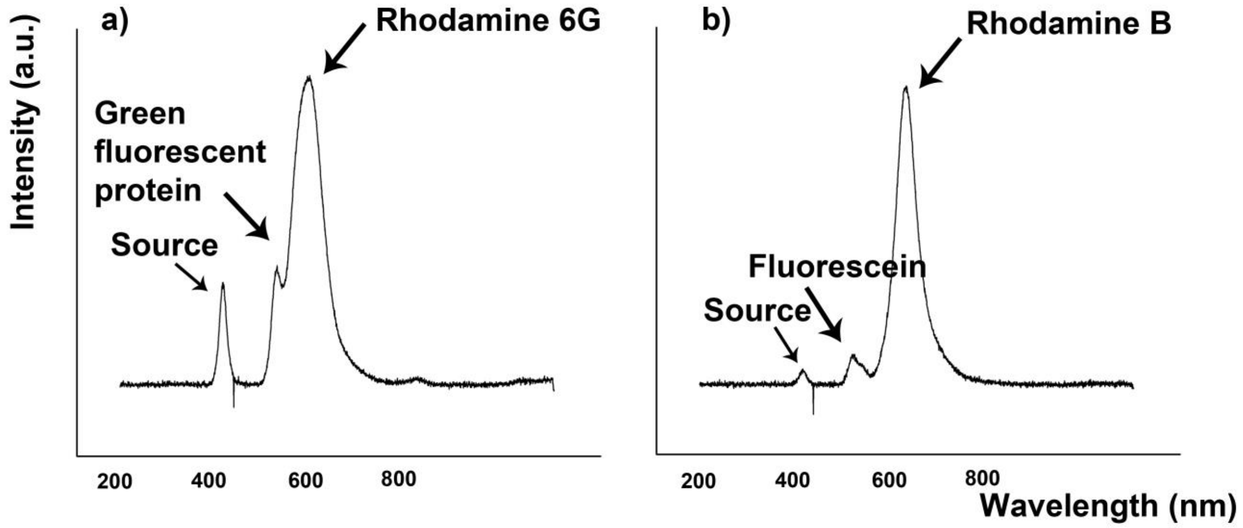

Figure S7 Spectra of light collected from fluorescent dyes. a) and b) different mixtures of fluorescent dyes excited by external light and emission signal was collected by the nanoendoscope.

Fluorescent Dye Experiments: Simultaneous Excitation and Collection:

Spectrum collection was done using simultaneous excitation and collection of emission signal using fluorescent dyes (Figure S8). Three commercially available dye solutions were used in this experiment. Endoscope was dipped into these solutions and excited with 532 nm light and emission was also connected and sent to a spectrometer using a Y-splitter as shown in the schematic Figure S8(a). The emitted spectra from the dyes (b) Bromofluroscene, (c) Rhodamine 6G and (d) Rhodamine B was collected by the same endoscope tip and analyzed using the attached spectrometer.

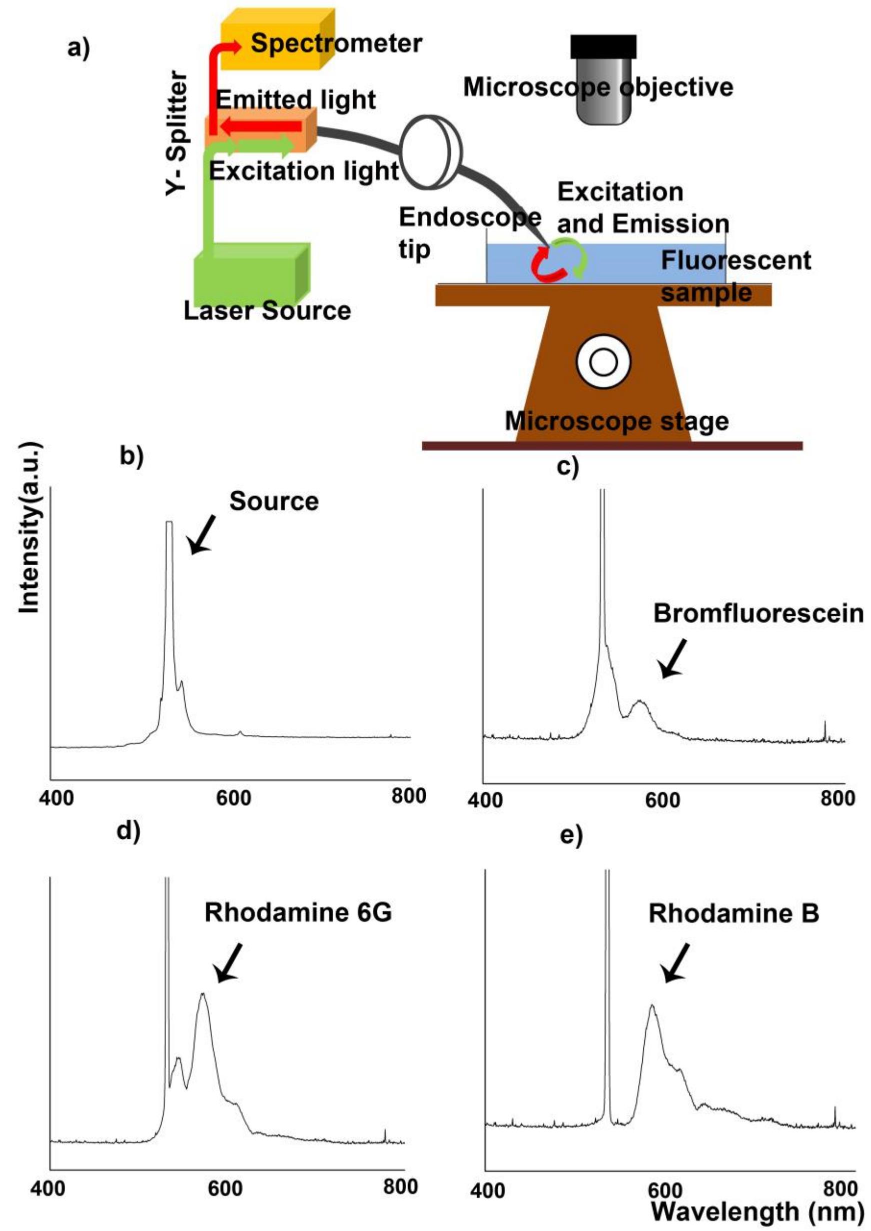

Figure S8 Simultaneous light excitation and collection of emission from fluorescent dyes. a) spectra of the source 532 nm and c-e various dyes used in this experiment.

Cell staining methods

Fibroblast Acridine Orange Experiment:

The fibroblast cells were fixed in in 4% paraformaldehyde, rinsed with phosphate buffer saline (PBS) solution and incubated for 2 minutes in Acridine Orange solution of concentration 10 μg/ml, and then were rinsed and used in nano-endoscopy experiments.

MDA-MB-231 Mitochondria Experiments:

Mitochondria were labeled by incubating cells with 100 nM MitoTracker (Life Technologies) at 37°C for 30 min followed by PBS washing for three times. Stained live cells were subjected to nanoendoscopy experiment.

Liver cell Hoechst Experiments:

Live cells were stained with Hoechst (10 μg/mL) diluted in HCM for 30 minutes at 37°C, rinsed and used in nano-endoscopy experiments.

Cell viability:

Experiment 1: Live Dead Cell kit showing green fluorescence of live cells continuously interrogated with the nano-endoscope.

Experiment 2: Live cells were stained with CellTracker™ Green CMFDA Dye (50 μg/mL) and Hoechst (10 μg/mL) diluted in HCM for 30 minutes at 37°C.

Experiment 3: Live cells were stained with CellTracker™ Green CMFDA Dye (50 μg/mL) and Hoechst (10 μg/mL) diluted in HCM for 30minutes at 37°C.

References:

1. Walling, M.A. and J.R.E. Shepard, "Cellular heterogeneity and live cell arrays". *Chemical Society Reviews*, 2011. **40**(7): p. 4049-4076.

2. Vo-Dinh, T. and P. Kasili, "Fiber-optic nanosensors for single-cell monitoring". *Analytical and Bioanalytical Chemistry*, 2005. **382**(4): p. 918-925.
3. Shambat, G., S.-R. Kothapalli, J. Provine, T. Sarmiento, J. Harris, S.S. Gambhir, and J. Vučković, "Single-Cell Photonic Nanocavity Probes". *Nano Letters*, 2013.
4. Orynbayeva, Z., R. Singhal, E.A. Vitol, M.G. Schrlau, E. Papazoglou, G. Friedman, and Y. Gogotsi, "Physiological validation of cell health upon probing with carbon nanotube endoscope and its benefit for single-cell interrogation". *Nanomedicine-Nanotechnology Biology and Medicine*, 2012. **8**(5): p. 590-598.
